# Loop underlay tympanoplasty for anterior, subtotal and total tympanic membrane perforations: a retrospective review

**DOI:** 10.1186/s40463-019-0335-x

**Published:** 2019-03-12

**Authors:** Rana Barake, Tamer El Natout, Marc Bassim, Mohammad Ali El Natout

**Affiliations:** 1 Department of Otorhinolaryngology - Head and Neck Surgery, American University of Beirut Medical Center, Riad El Solh, Beirut, 1107 2020 Lebanon; 2 Royal College of Surgeons in Ireland, 123 St Stephen’s Green, Dublin 2, Ireland

**Keywords:** Tympanic membrane perforation, Tympanoplasty, Underlay tympanoplasty, Conductive hearing loss

## Abstract

**Background:**

The study aims at reporting our experience with loop underlay tympanoplasty, a modification of the underlay technique previously reported, for the reconstruction of anterior, subtotal or total tympanic membrane perforations.

**Methods:**

A retrospective review of charts of patients who have undergone loop underlay tympanoplasty from January 2002 to January 2012 was performed. One thousand one hundred patients were included. Hearing test results preoperatively and postoperatively were reported. On follow up visits, the closure of the tympanic membrane perforation and the improvement of hearing compared to preoperative measurements with absence of complications were considered as successful outcomes of the surgery.

**Results:**

At the three-month follow-up visit, the perforation closure rate was found to be 99.3% and Air-Bone Gap closure rate to less than 10 dB was 99.5%. The complication rate, including post-operative infection, was 0.72%.

**Conclusion:**

The loop underlay technique combines advantages of both underlay and overlay techniques with excellent postoperative outcomes.

## Background

Type I tympanoplasty involves the repair of tympanic membrane perforations in the presence of an intact ossicular chain and would result ultimately in normal postoperative hearing results [1].

Many techniques and modifications have been developed for the repair of tympanic membrane perforations and can generally be divided into the underlay or overlay grafting techniques, which involve, respectively, the insertion of a graft either medially or laterally to the fibrous tympanic membrane annulus [2, 3]. Each technique has its own application, advantages and complications.

In this study, we introduce the loop underlay tympanoplasty, a novel modified underlay technique with a superiorly based skin flap, for the reconstruction of anterior, subtotal or total tympanic membrane perforations. This technique combines the ease of the underlay technique, with the higher success rate of the overlay approach for this kind of perforations. Thus, we hypothesize that the loop underlay tympanoplasty is superior to both in terms of perforation closure rate and closure of air-bone gap.

Our aims are to present the loop underlay grafting surgical technique and to evaluate its success rate in a large patient series.

## Methods

This study was approved by the Institutional Review Board at the American University of Beirut and at Hammoud Medical Center. The outcomes of all patients who underwent loop underlay tympanoplasty at the American University of Beirut Medical Center and the Hammoud Medical Center from January 2002 until January 2012 were retrospectively reviewed. A total of 1194 charts were reviewed and 94 charts were excluded due to exclusion criteria. Inclusion criteria included patients with any cause of anterior, subtotal or total tympanic membrane perforation, who underwent loop underlay tympanoplasty with follow-up visits for a minimum of 3 months postoperatively. Exclusion criteria included any patient with posterior perforation, active ear infection and otorrhea, ossicular chain disease or cholesteatoma. The type of tympanic membrane perforation was determined by checking the documented diagnosis in the patient’s chart. Anterior perforation was defined as any perforation anterior to the malleus. Total perforations are those in which there is only a minimal rim of membrane left around the annulus and along the malleus handle while subtotal perforations are those larger than 50% but smaller than total perforations.

Charts were reviewed for demographic data as well as the following surgical outcomes: healing of the tympanic perforation, postoperative hearing test results, and the incidence of complications. The patients were examined at least 3 months after surgery with repeat audiograms, but the outcomes were compared for all patients at the 3-month-visit. Hearing test results preoperatively and postoperatively were reported using three-frequency (500, 1000 and 2000 Hz) air-bone gaps and tabulated for comparison as less than or equal to ten decibels, eleven to twenty decibels, twenty-one to thirty decibels and more than thirty decibels. Possible complications included infection, blunting or graft lateralization, and worsening of the hearing impairment. On follow up visits, the closure of the tympanic membrane perforation, the absence of infection, blunting or graft lateralization, and the improvement of hearing to less than 10 dB air-bone gap compared to preoperative measurements were considered as successful outcomes of the surgery.

### Surgical technique

**Fig. 1:**
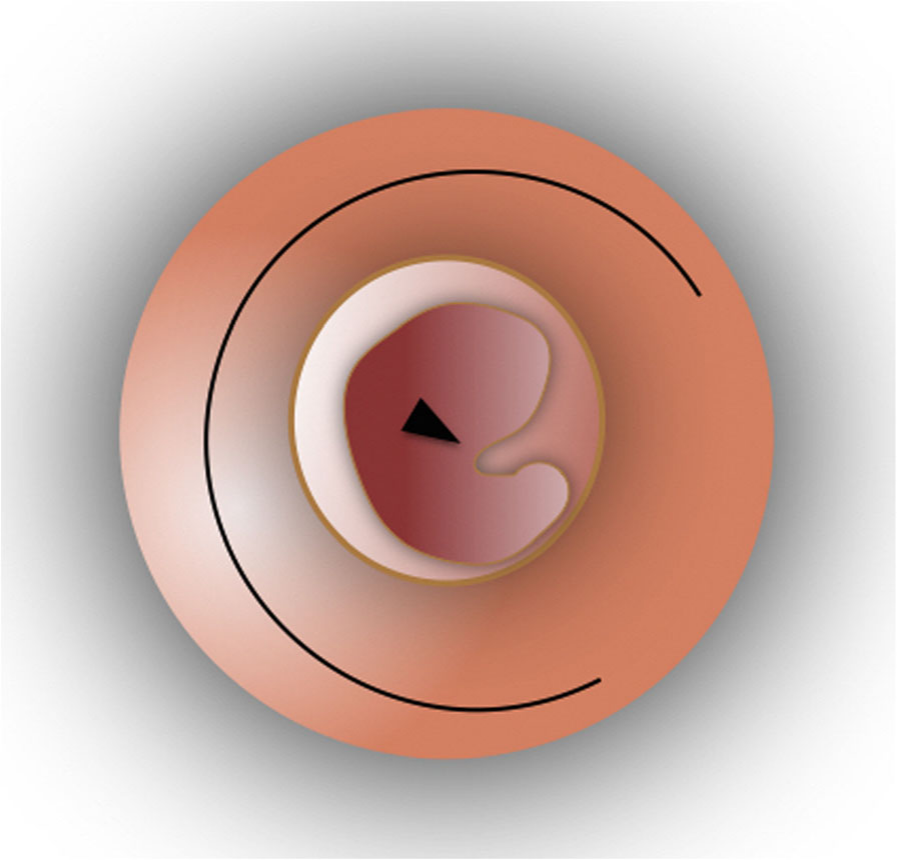
Circumferential canal skin incision is performed from the 11 o’clock position to the 2 o’clock position passing through the 6 o’clock position. Black arrowhead pointing towards the malleus

**Fig. 2:**
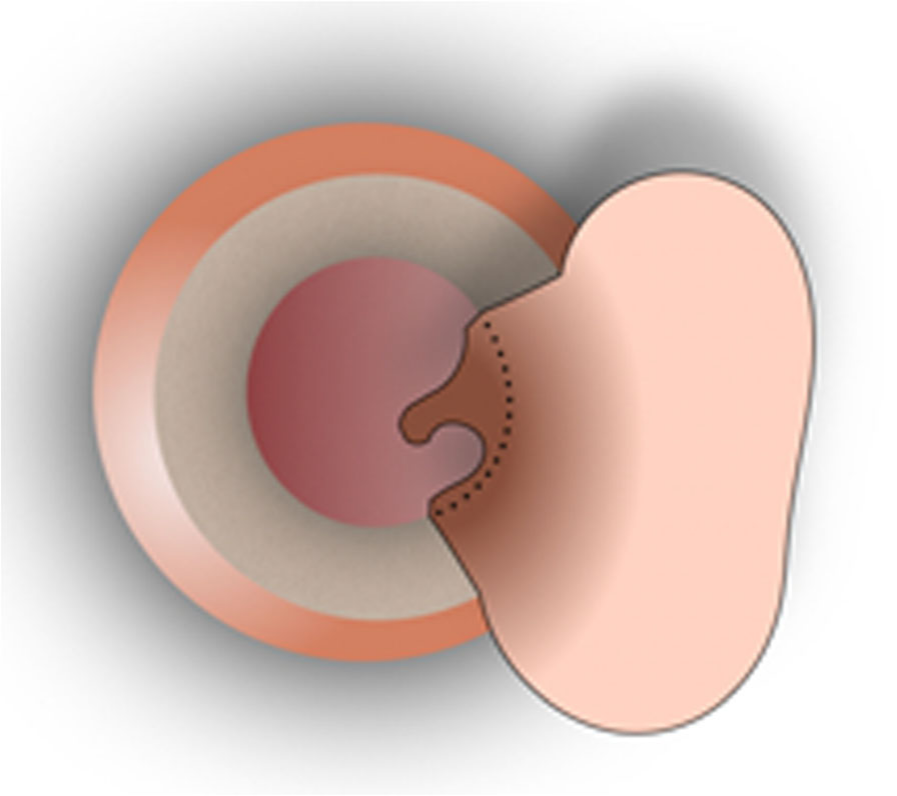
The superiorly based “loop” tympanomeatal flap including all three layers of the remnant tympanic membrane is elevated along with the fibrous annulus

**Fig. 3:**
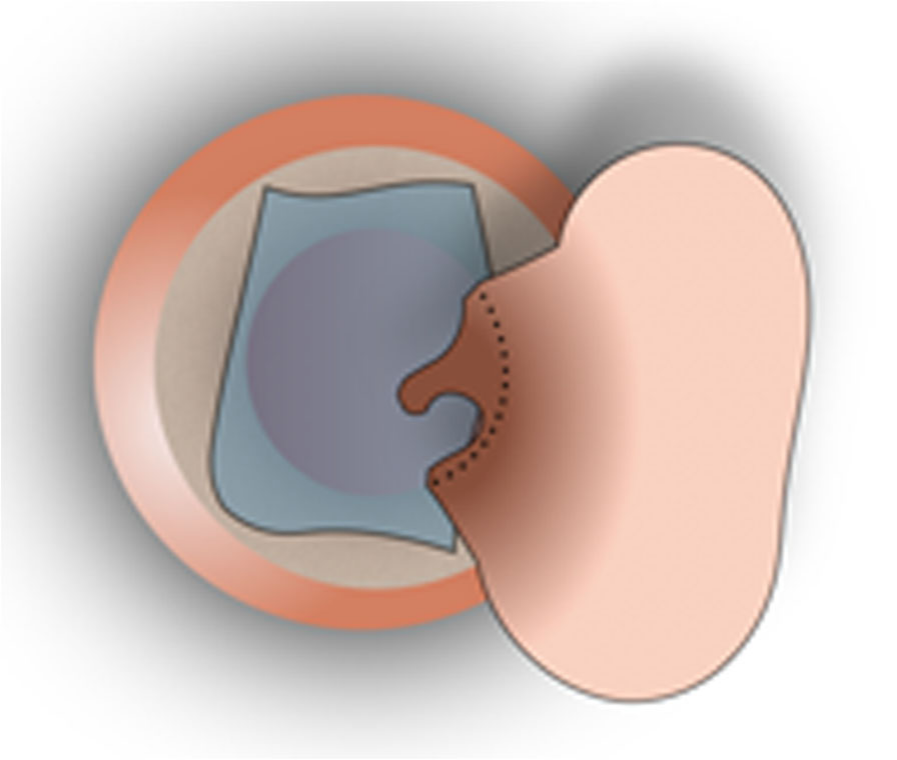
The temporalis fascia graft is placed medial to the fibrous annulus and the malleus handle, laying over the bony ear canal anteriorly, inferiorly and posteriorly

**Fig. 4:**
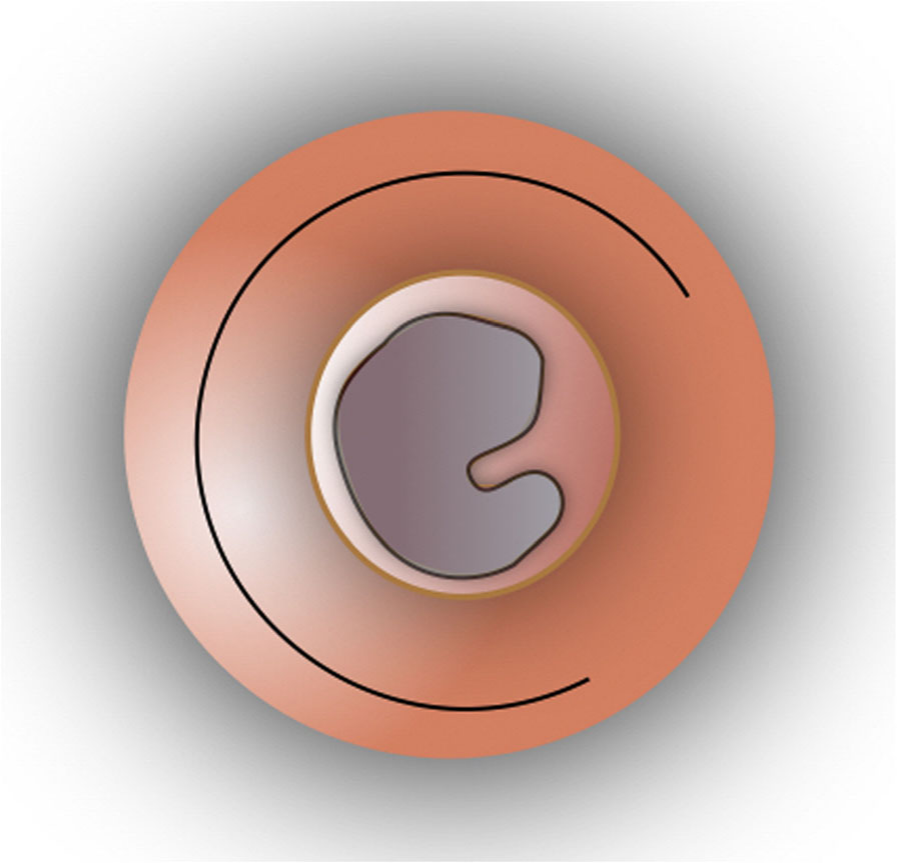
The “loop” tympanomeatal flap is repositioned back in place

The surgery is performed under general anesthesia through a typical postauricular incision. Temporalis fascia graft is harvested followed by accessing the auditory canal and freshening the edges of the tympanic membrane perforation. Then, a circumferential canal skin incision is performed 1 cm away from the annulus, from the 11 o’clock position to the 2 o’clock position passing through the 6 o’clock position. Then, the superiorly based “loop” shaped tympanomeatal flap including all three layers of the remnant tympanic membrane is elevated along with the annulus fibrosus which is elevated from the bony sulcus (Figs. 1 and 2). The skin flap is then pedicled superiorly on the malleus remnant. The graft is then placed on the bony canal anteriorly, inferiorly and posteriorly medial to the malleus and medial to the annulus remnant, which was elevated. Finally, the tympanomeatal flap is repositioned (Figs. 3 and 4).

The temporalis fascia graft is supported medially by packing the middle ear and laterally by packing the ear canal with Gelfoam (Pharmacia & Upjohn Co., Kalamazoo, MI, USA).

Post-operatively, the patients are typically seen first time at week one to examine the post-auricular wound and started on antibiotic eardrops. The patients are then seen at week two for Gelfoam packing removal. Patients are then seen monthly. No touch-ups are performed at follow-ups.

The temporalis fascia is used as a graft material in our technique. It is soft, pliable, can cover the large exposed surfaces of the canal walls in addition to the large sized perforations, and is widely used in tympanoplasties. It is also easily accessible through the post-auricular incision. Harvesting perichondrium would have added the morbidity of a second incision. Cartilage is not commonly used in our group for tympanoplasties.

In cases where the annulus ligament is absent, the technique applied is the same while making sure to fully elevate the skin anteriorly, taking as a safe margin a sliver of the mucosa of the Eustachian Tube, thus making sure we don’t err on the side of a short flap.

Only few cases were combined with canaloplasty as the technique provides excellent visualization of the middle ear without the need for drilling in most cases.

## Results

The medical records of a total number of 1100 patients who had undergone loop underlay tympanoplasty for anterior, subtotal and total tympanic membrane perforations were reviewed. Six hundred sixty five (60.5%) patients were males and 435 (39.5%) patients were females. Three hundred and thirty (30%) patients had an anterior tympanic membrane perforation, whereas 780 (70%) had a subtotal or total perforation. Eight hundred and ten cases were primary whereas 290 cases were revision surgeries (Table 1).

**Table 1:** Distribution of cases as per type of perforation and type of surgery

	Type of Surgery	
Type of Perforation	Primary (*n* = 810)	Revision (*n* = 290)
Total Perforation (*n* = 260)	155	105
Subtotal perforation (*n* = 520)	470	50
Anterior Perforation (*n* = 330)	195	135

The patients were examined several months after surgery with repeat audiogram. Tympanic membrane closure was assessed as well as presence of any complication.

The success rate or rate of complete closure of the tympanic membrane perforation on 3-month follow-up was found to be 99.3% (1092 of 1100). All patients had complete closure of their tympanic membrane perforation on follow-up except 8 patients who showed residual perforation. This postoperative complication was due to postoperative infection along with concurrent otorrhea in all the concerned patients. None of the patients had lateralization of the graft. Few cases ended up with anterior blunting which did not affect audiological outcome.

The preoperative and most recent postoperative audiogram air-bone gaps at the three frequencies 500 Hz, 1000 Hz, and 2000 Hz were tabulated (Table 2). The proportion of patients who had an average air-bone gap of less or equal to 10 dB was 0% preoperatively vs. 99.5% postoperatively.

**Table 2:** Comparison of preoperative and postoperative pure tone audiometry air-bone gaps

	Preoperative Air-Bone Gap (*N* = 1100) (%)				Postoperative Air-Bone Gap (N = 1100) (%)			
Frequency	≤10 dB	11–20 dB	21–30 dB	≥30 dB	≤10 dB	11–20 dB	21–30 dB	≥30 dB
500 Hz	0 (0)	65 (6)	785 (71)	250 (23)	1095 (99.5)	5 (0.5)	0 (0)	0 (0)
1000 Hz	0 (0)	50 (5)	820 (74)	230 (21)	1095 (99.5)	5 (0.5)	0 (0)	0 (0)
2000 Hz	0 (0)	50 (5)	800 (72)	250 (23)	1095 (99.5)	5 (0.5)	0 (0)	0 (0)

## Discussion

Classically, tympanic membrane perforations have been repaired using either the underlay or the overlay techniques. Each technique has its own advantages and disadvantages. The most commonly performed technique is the underlay tympanoplasty where the graft is placed medial to the annulus and the malleus [2]. It is frequently used in posterior perforations. It is easier to perform and has a high success rate. However, in anterior perforation repair, it has a higher risk of fall of the anterior portion of the fascia graft due to lack of anterior support with subsequent failure of closure and partial obliteration of the middle ear cavity [4]. In the overlay grafting technique, first described by Shea in 1960 [5] then modified by Sheehy and Glasscock in 1967 [6], the graft is placed lateral to the annulus, on top of the fibrous layer of the tympanic membrane remnant. It is reported to be more successful in anterior and subtotal perforations, however, it is more technically challenging. It provides a wide exposure and high take rate but has a significantly higher risk of graft lateralization [4], blunting and canal stenosis, impaired vascular supply and delayed healing, as well as a higher risk of cholesteatoma formation than the underlay technique.

According to the literature, the most challenging tympanic membrane perforations are the anterior or anterosuperior tympanic membrane perforations because of access difficulty, limited blood supply, and lack of support to the graft, keeping in mind the importance of surgeon experience in such perforations, hence the continuous search for the optimal technique for improved outcomes [4]. Some of the technique modifications published so far for that cause include the three-point fix technique [7], loop overlay tympanoplasty with a superiorly based tympanomeatal flap and overlay placement of the graft [8], sandwich graft tympanoplasty [9], over-under tympanoplasty where the graft is placed over the malleus and under the annulus [10], and mediolateral graft tympanoplasty [11] among others.

In this study, we introduced the novel loop underlay technique, which involves performing a superiorly based tympanomeatal flap thus maintaining its superior attachment to the ear canal and preserving the posterosuperior blood supply. This will promote better healing of the tympanic membrane perforation and better take rate of the graft. The position of the fascia on the anterior canal wall, under the annulus, prevents medialization of the graft, which is often the mechanism of failure in anterior perforations. The graft design is skewed and extended 5 mm anterosuperior to allow covering of the bony canal and preventing medialisation in the area. In addition, the temporalis fascia graft is placed in an underlay fashion, which avoids graft lateralization and blunting of the external auditory canal.

In the most recent comparative study published in 1997, Rizer showed that the overlay technique was superior to the underlay technique with fewer complications and a higher success rate of 95.6% versus 88.8% for the underlay technique [12]. This contradicted with previous studies by Glasscock and Doyle showing a superiority of the underlay technique due to easier technique and shorter healing time as compared to the overlay technique [13, 14]. Rizer attributed his results mostly to a good surgical technique and experience as well as a good exposure especially when a postauricular approach and canaloplasty are performed [12]. In the loop underlay tympanoplasty, the approach is through a postauricular incision hence a better access to the tympanic membrane perforation and an easier technique, and it requires less surgical skills than one that involves overlay placement of the tympanic membrane. Our success rate of 99.3% exceeds the previously reported underlay and overlay graft take rates. Some of the main technical challenges of this technique are the need for a meticulous dissection of skin anteriorly, the need to make sure that the skin side of the flap is properly oriented upon repositioning, and the need to properly position the graft to avoid blunting anteriorly, especially when no canaloplasty is done.

Our study was limited by the variable follow-up period in such a large patient population; hence the fixed 3-month visit was chosen as the reference point for outcome assessment. This limits our assessment of possible post-operative complications at longer follow-up periods, including possible reperforations and cholesteatoma formation. Another weakness is the retrospective nature of the study limiting us because of the inadequacy and inaccuracy of documentation in the patient charts, especially since hospital documentation from 2002 to 2012 was not computerized, not standardized. Further prospective studies with longer follow-up periods would be recommended.

## Conclusion

The loop underlay technique combines both underlay and overlay technique advantages with excellent postoperative outcomes, having a success rate of 99.3% and very minimal rate of complications and we recommend it for the reconstruction of anterior, subtotal and total tympanic membrane perforations.
